# Long-term dominance of *Mycobacterium tuberculosis* Uganda family in peri-urban Kampala-Uganda is not associated with cavitary disease

**DOI:** 10.1186/1471-2334-13-484

**Published:** 2013-10-17

**Authors:** Eddie M Wampande, Ezekiel Mupere, Sara M Debanne, Benon B Asiimwe, Mary Nsereko, Harriet Mayanja, Kathleen Eisenach, Gilla Kaplan, Henry W Boom, Sebastien Gagneux, Moses L Joloba

**Affiliations:** 1 Department of Medical Microbiology, College of Health Sciences, Makerere University, Kampala, Uganda; 2 Department of Pediatrics and Child Health College of Health Sciences, Makerere University, Kampala, Uganda; 3 Department of Epidemiology and Biostatistics, School of Medicine, Case Western Reserve University, Cleveland, OH, USA; 4 Uganda-Case Western Reserve University Research Collaboration, Kampala, Uganda; 5 Department of Pathology, University of Arkansas for Medical Sciences, Little Rock, AR, USA; 6 Laboratory of Mycobacterial Immunity and Pathogenesis, Public Health Research Institute, University of Medicine and Dentistry, Newark, NJ, USA; 7 Tuberculosis Research Unit, School of Medicine, Case Western Reserve University and University Hospitals of Cleveland, Cleveland, OH, USA; 8 Department of Medical Parasitology and Infection Biology, Swiss Tropical and Public Health Institute, Basel, Switzerland; 9 University of Basel, Basel, Switzerland; 10 Department of Bio-molecular Resources and Biolab Sciences, College of Veterinary Medicine, Animal Resources and Bio Security, Makerere University, Kampala, Uganda; 11 Department of Medical Microbiology, School of Biomedical Sciences, College of Health Sciences, Makerere University, P.O BOX 7072, Kampala, Uganda

**Keywords:** *Mycobacterium tuberculosis complex*, Lineage, Single nucleotide polymorphism, Mycobacteria, Strain family, Cavitation, Virulence, Epidemiology, Evolution

## Abstract

**Background:**

Previous studies have shown that *Mycobacterium tuberculosis* (MTB) Uganda family, a sub-lineage of the MTB Lineage 4, is the main cause of tuberculosis (TB) in Uganda. Using a well characterized patient population, this study sought to determine whether there are clinical and patient characteristics associated with the success of the MTB Uganda family in Kampala.

**Methods:**

A total of 1,746 MTB clinical isolates collected from1992-2009 in a household contact study were genotyped. Genotyping was performed using Single Nucleotide Polymorphic (SNP) markers specific for the MTB Uganda family, other Lineage 4 strains, and Lineage 3, respectively. Out of 1,746 isolates, 1,213 were from patients with detailed clinical data. These data were used to seek associations between MTB lineage/sub-lineage and patient phenotypes.

**Results:**

Three MTB lineages were found to dominate the MTB population in Kampala during the last two decades. Overall, MTB Uganda accounted for 63% (1,092/1,746) of all cases, followed by other Lineage 4 strains accounting for 22% (394/1,746), and Lineage 3 for 11% (187/1,746) of cases, respectively. Seventy-three (4 %) strains remained unclassified. Our longitudinal data showed that MTB Uganda family occurred at the highest frequency during the whole study period, followed by other Lineage 4 strains and Lineage 3. To explore whether the long-term success of MTB Uganda family was due to increased virulence, we used cavitary disease as a proxy, as this form of TB is the most transmissible. Multivariate analysis revealed that even though cavitary disease was associated with known risk factors such as smoking (adjusted odds ratio (aOR) 4.8, 95% confidence interval (CI) 3.33-6.84) and low income (aOR 2.1, 95% CI 1.47-3.01), no association was found between MTB lineage and cavitary TB.

**Conclusion:**

The MTB Uganda family has been dominating in Kampala for the last 18 years, but this long-term success is not due to increased virulence as defined by cavitary disease.

## Background

Globally there are seven human-associated *Mycobacterium tuberculosis* complex (MTBC) lineages that are differentially distributed with certain lineages predominating in certain geographical regions and human populations [1-4]. Increasing evidence shows that these lineages differ in pathogenesis in animal models, but their differential impact on tuberculosis (TB) in humans is not clear [3]. There is also inconclusive data with respect to whether the distribution of MTBC lineages/sublineages is due to host and or microbial factors [3,5,6]. Recent studies in Uganda indicated that the majority of TB cases are due to the MTBC Uganda family (L4-U) [7,8], a sub-lineage of Lineage 4 defined by a deletion in the Region of Difference (RD) 724, the spoligotype finger print (33–36, 40 and 43 spacers missing), and several SNPs [1,9,10]. Although earlier studies had defined this L4-U family as *Mycobacterium africanum* sub-type II based on colony morphology and biochemical tests [11,12], advances in molecular classification have led to its reclassification as *M. tuberculosis* sensu stricto [13].

The resurgence of TB calls for improved understanding of the epidemiology, pathogenesis, chemotherapy, and genetic variability of the causative agent for better control of the disease. Studies so far offer limited information about the kinetics of L4-U, and do not explain why this family of MTBC is so predominant in Uganda. However, it is now apparent that host, environment and microbiological factors are likely to play a role [2,9,14-22]. For instance, the dominance of Lineage 2 (which includes the Beijing family of MTBC) in Asia and its wide geographical distribution might be partially due to higher virulence (as determined in animal models) and its association with drug resistance [23-25]. Furthermore, based on the long-standing association between MTBC and its human host, some studies have proposed that the different MTBC lineages might have adapted to different human populations, perhaps as a consequence of co-evolutionary processes [1,6,23,26-28].

With the advent of robust molecular markers and a well characterized large human population cohort, genetic variability in MTBC clinical isolates and clinical phenotypes can be better described, and thus the reason of dominance of certain MTBC lineages may be deduced [3]. In this study we used MTBC isolates collected from patients participating in two large prospective community-based TB transmission studies carried out in peri-urban Kampala from 1992–2009 to establish trends in the prevalence of the various MTBC lineages over time, and examine the association of MTBC lineages with patient characteristics.

## Methods

### Patient recruitment and collection of MTBC isolates

The isolates used in this study were collected from patients recruited in two studies that were both carried out in peri-urban Kampala-Uganda in sequence. An initial household contact study (HC) was conducted from 1992 to 1999 to describe the epidemiology of TB [population 1.7 million; population density 9400/km^2^ (Uganda Bureau of Statistics; http://www.ubos.org, 2011) and [29,30]. The second study is the Kawempe Community Health study (KCH) that started in 2000 and is ongoing. The KCH focuses on host factors associated with primary infection, re-infection, reactivation, and progression from MTB infection to active TB clinical disease and also identifies and tracks strains of MTB circulation in households and the local community.

During the conduct of these epidemiologic cohort studies (HC and KCH), adults with sputum smear positive TB were consecutively enrolled as index cases. An index case was defined as the first TB case identified in a household who was ≥ 18 years of age and lived with one or more household contacts. A household was defined as a group of people living within one residence, share meals together and identified a head of family who made decisions for the household. Following the identification of the index cases, home health visitors contacted the household contacts for health education about TB and the study. A household contact was defined as any individual who had resided in the household for at least 7 consecutive days during the 3 months prior to the diagnosis of TB in the index case. Household contacts were screened for both latent (tuberculin skin test) and active disease (sputum smear and culture) on first contact. Those found not to have TB according to the study protocol were followed every three months for a period of two years to identify contacts that later developed active TB. Household contacts were classified as co-prevalent cases if active TB was present at baseline or during three months of household follow-up and as incident cases if active TB developed after three months of follow-up. In both studies (HC and KCH) a total of 1746 isolates were stored from the study area over the period of study (1995–2009). Patients with either latent or active TB were treated with isoniazid (INH) preventive therapy or standard short course combination chemotherapy for active TB in accordance with the Uganda National TB and Leprosy Program guidelines.

At baseline, data of enrolled patients, including age, sex, HIV status, presence of cavity, ethnicity, status of smoking, Body Mass Index (BMI), level of education, alcohol drinking, income, history of diabetes, presence of BCG scar, night sweats, TB in the past, hemoptysis, swollen lymph nodes, extent of disease on chest radiographs and smear grade, were recorded. The extent of disease on chest radiographs was classified as normal, mild, moderate, or far advanced using a validated, standardized scheme [31], with lesions recorded by an independent reader who was blinded to smear and culture results. Sputum smear microscopy and culture were performed at either the National TB Reference Laboratory (NTRL) or the Joint Clinical Research Centre (JCRC) TB Laboratory. Isolates were confirmed as MTB using the BACTEC^®^ para-nitro-acetyl amino-hydroxy-propiophenone (NAP) susceptibility method [32] and later stored at – 80°C in 7H9 broth supplemented with OADC and glycerol for future analyses.

The institutional review boards and ethics committees at Case Western Reserve University, Makerere University, and the Ugandan AIDS Research Council, and the Uganda National Council for Science and Technology approved the study protocols. All patients gave written informed consent for study participation, including pre- and post-HIV test counseling.

### Extraction of MTBC genomic DNA from stored isolates

A total of 1,746 isolates were stored in replicates at either JCRC or NTRL laboratories at – 80°C. Isolates corresponding to an individual patient were selected for genotyping. To extract DNA, the selected isolates were thawed overnight at – 20°C and later at room temperature for 12 h. The vials were centrifuged at 15,000 g for 30 min and the pellet washed twice with 500 μl of Qiagen PCR water. The final pellet was re-suspended in 100 μl of Qiagen PCR-water, heated at 95°C for 30 minutes to kill and lyse the bacilli and later sonicated for 15 min at room temperature. The extracted genomic DNA in the supernatant was recovered by centrifugation at 15,000 g for 30 min; the latter was used immediately in the real time PCR (RT-PCR) assay or stored at -20°C for future use.

### Genotyping MTBC isolates with Single Nucleotide Polymorphic (SNP) markers by RT-PCR assay

To ascertain the lineage of an MTB isolate, RT-PCR (Roche Light Cycler^®^ 480) was performed using specific primers and hybridization probes (HyProbe, FRET probes) containing SNPs designed based on the work published by Comas *et al.*[9] and Hershberg *et al.*[10] (See Additional file 1: Table S1). RT-PCR analysis involved, amplification (40 cycles of 95°C for 10s, 57/53/51°C for 10s and 72°C for 10s) of the target region(s) to generate amplicons for melting curve analysis. Melting curves were analyzed using lightCycler^®^ software version 1.5 to assign an isolate to a particular lineage depending on the melting temperature (Tm) at which the hybridization probes dissociates from the amplicons. For validation purposes, Long Sequence Polymorphism PCR (LSP-PCR) analysis was performed as previously described [1,20]. In all the assays, we used MTB L4-U genomic DNA from our laboratory, H37Rv genomic DNA (Lineage 4) and Lineage 3 (Central Asian strain) genomic DNA (Courtesy of Mark Nicol) as positive control DNA.

### Identification of MTBC lineages in the clinical isolates collected from peri-urban Kampala

A total of eight (8) SNPs, 3 for identifying MTB Uganda (L4-U): (Rv0006_0238n, Rv0040c-0619n and Rv2949c-0375s); 2 for MTB Lineage 4: (Rv 0407-0960s and Rv 2962c-0711) and then 3 for MTB Lineage 3: (Rv 0129c_0472n, Rv 2959c-0219n and Rv 3133c-0419) each with its accompanying designed primers and probes were optimized for use in RT-PCR SNP assays to identify MTBC lineages on the basis of differences in melting temperature (Tm). Since the SNPs identifying each lineage were mutually exclusive, we selected a single SNP from each lineage, which provided reliable results, for genotyping the 1,746 MTB isolates. For the Uganda family sub lineage we selected 2 SNPs, one for identifying Uganda I and the other for Uganda II MTB (See Additional file 1: Table S1). Genotyping results based on the designed RT-PCR SNP assay were consistent with that based on long sequence polymorphism (LSP) (data not shown).

### Statistical analysis

Patient characteristics were compared using the chi-square test for binary data and Student’s t-test for continuous variables. A series of univariate and multivariable logistic regression models were fitted to evaluate the relationship between MTB lineage (primary independent variable) and severity of TB disease on chest radiograph (cavitary TB) or smear status (dependent variable). Lung cavitation as a radiographic variable was used as a measure for severity of disease since it is associated with worse symptoms of TB [33-35]. Age, sex, HIV status and other patient characteristics were used as covariates (See Table 1).

**Table 1:** Distribution of patient variables across the 3 main MTB lineages in peri-urban Kampala

**Variable**	**Category**	**Patients characteristics (n, %)**	**L4-U (n=788)**	**L4-NU (n=289)**	**L 3 (n=136)**	P-value*
Age^1^	> 30 years	757 (69)	503 (70)	173 (66)	81 (68)	0.44
	≤ 30 years	340 (31)	213 (30)	89 (34)	38 (32)	
Sex^2^	Female	503 (46)	325 (45)	120 (46)	58 (49)	0.79
	Male	594 (54)	391 (55)	142 (54)	61 (51)	
HIV status^3^	Negative	669 (65)	450 (66)	157 (62)	62 (60)	0.29
	positive	367 (35)	230 (34)	95 (38)	42 (40)	
Cavity^4^	No	441 (47)	296 (48)	106 (48)	39 (39)	0.25
	Yes	500 (53)	324 (52)	115 (52)	61 (61)	
Ethnicity^5^	Non-bantu	64 (6)	39 (6)	14 (6)	11 (9)	0.27
	Bantu	984 (94)	641 (94)	238 (94)	105 (91)	
Smoking^6^ status	Never smoked	659 (62)	443 (65)	155 (60)	61 (52)	0.03
	Current or ever smoked	401 (38)	243 (35)	102 (40)	56 (48)	
BMI^7^	Weight loss	557 (49)	374(50)	121(44)	62(48)	0.19
	No loss	588 (51)	368(50)	154(56)	66(52)	
Level of education^8^	Low	377 (35)	248 (36)	93(36)	36(31)	0.52
	High	688 (65)	444(64)	163(64)	81(69)	
Drinking alcohol^9^	Yes	162 (21)	106 (21)	40(21)	16(22)	0.99
	No	611 (79)	402(79)	151(79)	58(78)	
Income^10^	Low	270 (46)	162(44)	69(46)	39 (61)	0.048
	High	314 (54)	203(56)	82 (54)	29 (39)	
diabetic Patients^11^	Yes	12 (1)	8(2)	3(2)	1(2)	0.97
	No	922 (99)	604(98)	224(98)	94(98)	
Presence of BCG scar^12^	Yes	756 (62)	488 (62)	183 (63)	85 (63)	0.92
	No	456 (38)	299 (38)	106 (37)	51 (38)	
Night sweets^13^	No	533 (47)	340(46)	133(49)	60 (47)	0.69
	Yes	608 (53)	401 (54)	139(51)	68 (53)	
TB in the past^14^	Yes	17 (2)	10 (2)	3 (2)	4 (4)	0.18
	No	953 (98)	619 (98)	239 (98)	95 (96)	
Hemoptysis^15^	No	173 (15)	111(15)	43(16)	19 (15)	0.95
	Yes	972 (85)	632(85)	231(84)	109 (85)	
Swollen lymph nodes^16^	No	46 (6)	26 (5)	16 (8)	4 (5)	0.26
	Yes	727 (94)	482(95)	175(91)	70 (95)	
Extent of lung involvement^17^	Normal/mild	733 (73)	475 (72)	180 (75)	78 (76)	0.58
	Advance/far advanced	271 (27)	185 (28)	61 (25)	25 (24)	
Smear grade^18^	≤10 AFB/field	227 (33)	146 (33)	65 (37)	16 (24)	0.1377
	>10 AFB/field	454 (67)	294 (67)	109 (63)	51 (76)	

*p-value obtained by chi-square statistic; 1 = 116 missed data for age, 2 = 116 missed data for sex, 3 = 177 missed data for HIV status, 4 = 272 missed data for cavity, 5 = 165 missed data for ethnicity, 6 = 153 missed data for smoking status, 7= 68 missed data for BMI, 8= 148 missed data for level of education, 9= 440 missed data for drinking alcohol, 10= 633 missed data for income, 11= 279 missed data for diabetes history, 12= 1 missed data for BCG status, 13 = 72 missed data for night sweating, 14= 243 missed data for history of TB, 15= 68 missed data for hemoptysis, 16 = 440 missed data for swollen glands, 17= 209 missed data for extent of lung involvement, 18=532 misses data of smear grade.

In order to predict the transmission dynamics over a span of 18 years, we performed a trend analysis using Poisson regression analysis with MTB lineage as the main predictor variable and adjustment for years. All analyses were performed using SAS version 9.2 (SAS Institute, Cary, NC).

## Results

### Overall and time-point prevalence of MTBC lineages in peri-urban Kampala from 1992–2009

**Figure 1:**
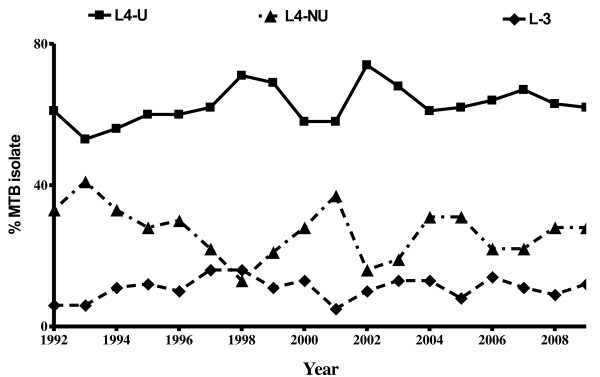
***M. tuberculosis *Uganda (L4-U) dominance over L4-NU and L3 has been stable between 1992–2009.** MTBC lineages were identified as described in the legend of Figure 1. Each data point [1992 (n=109), 1993 (n=71) 1994 (n=94), 1995 (n=73) 1996 (n=80), 1997 (n=82), 1998 (n=42), 1999 (n=63) 2000 (n=88), 2001 (n=68) 2002 (n=151), 2003 (n=148), 2004 (n=87), 2005 (n=119) 2006 (n=70), 2007 (n=132) 2008 (n=102), 2009 (n=94)] represent proportions of MTBC lineages observed per year.

In a RT-PCR SNP genotyping assay, a total of 1,746 MTBC isolates, each from a different TB patient, were analyzed. Overall, 63% (1092/1,746) were L4-U, 22% (394/1,746) were other Lineage 4 strains [from now onwards these shall be referred to as Lineage 4 non Uganda (L4-NU)], and 11% (187/1,746) Linage 3 (L3). The remaining 73 (4%) strains could not be classified based on the genotyping techniques used (Additional file 2: Figure S1). Next, we determined the point prevalence of the three predominant MTBC lineages during the previous 18 years (1992 to 2009). The data suggest that there was no trend over time for any MTBC lineage (Additional file 3: Figure S2). However, there were significant differences in MTBC lineage frequency over time (Poisson regression analysis, P<0.0001), with L4-U having the highest frequency during the whole study period, followed by L4-NU and L3. The difference in frequencies remained significantly different over the years examined (Figure 1). To explore the possible underlying basis for the long-term success of L4-U compared to the other lineages circulating in Kampala, we compiled and analyzed detailed clinical data.

### Patient characteristics across MTBC lineages

Of the 1,746 MTBC isolates we genotyped initially, we excluded 533 (31%) for this part of the analysis because they lacked patient information or were of unknown lineage. Hence, a total of 1,213 (69%) isolates remained corresponding to one TB patient each. The detailed patient characteristics of all of these patients are listed in Table 1.

Our univariate analysis taking lineage as the outcome showed that the proportions of patients infected with each MTBC lineage did not differ according to age, sex, HIV/TB co-infection, ethnicity, BMI, level of education, taking alcohol, history of diabetes, presence of BCG scar, night sweats, history of TB in the past, hemoptysis, swollen glands, extent of lung involvement or smear grade status (Table 1). However, we found that the proportion of TB patients with cavitary disease differed by MTBC lineage; however, this difference was not statistically significant (Table 1). Since cavitary disease is positively correlated with transmission and thus a measure of “virulence” [36], we decided to further investigate this difference, while controlling for the additional variables associated with cavitary TB.

### Patient risk factors associated with cavitary TB in peri-urban Kampala

Our univariate analysis taking cavitary TB as the outcome confirmed that the difference between MTBC lineages was not statistically significant. But other factors were significantly associated with cavitary TB and included a history of smoking (OR 4.78; 95% CI 3.41-6.69), low income (OR 2.13, 95% CI 1.52-2.97 and hemoptysis (OR 2.10 95% CI 1.46-3.03 (Table 2). Our multivariable logistic regression analysis with MTBC lineage as the main predictor and after adjusting for age, HIV status, history of smoking, income of the patients, and hemoptysis, again confirmed that the odds of having cavitary TB did not differ significantly between the 3 MTBC lineages (Table 3). The factors independently associated with cavitary TB included smoking (adjusted odds ratio (aOR) 4.76, 95% CI 3.33-6.84), low income (aOR 2.10, 95%CI 1.47-3.01), signs of hemoptysis (aOR 1.64, 95%CI 1.10-2.42) and HIV status (aOR 0.62 95% CI 0.45-0.84 for HIV-positive patients) (Table 3).

**Table 2:** A**ssociation between MTB lineages and patient characteristics with cavitary TB**

**Variable**	**Category**	**Odds Ratio**^**©**^	**95% CI**	*P-value
Lineage	Lineage L4-NU	1	-	-
	Uganda (L4-U)	0.90	0.69-1.18	0.45
	Lineage 3	1.43	0.94-2.19	0.10
Age	> 30 years	1		
	≤ 30 years	1.13	0.85-1.49	0.40
Sex	Female	1		
	Male	1.24	0.96-1.60	0.10
HIV status	Negative	1		
	Positive	0.78	0.596-1.03	0.08
Smoking status	Never smoked	1		
	Current or ever smoked	4.78	3.41-6.69	<0.0001
Income	High	1		
	Low	2.13	1.52-2.97	<0.0001
Hemoptysis	Yes	1		
	No	2.10	1.46-3.03	<0.001

*p-value obtained by logistic regression analysis; **©**Unadjusted OR = Odds ratio.

**Table 3:** Multivariate analysis determining independent risk factors for development of cavitary TB

**Variable**	**Category**	**Odds ratio^®^**	**95% CI**	P-value*
Lineage	Lineage L4-NU	1	-	-
	Uganda (L4-U)	1.08	0.78-1.51	0.6422
	Lineage 3	1.32	0.78-2.22	0.296
Age	> 30 years	1	-	-
	≤ 30 years	0.866	0.63-1.20	0.38
HIV status	Negative	1	-	**-**
	Positive	0.62	0.45-0.84	0.0023
Smoking status	Never smoked	1	-	-
	Current or ever smoked	4.76	3.33-6.84	<0.0001
Income	High	1		
	Low	2.10	1.47-3.01	<0.0001
Hemoptysis	No	1	-	-
	Yes	1.64	1.10-2.42	0.014

*p-value obtained by logistic regression analysis; **^®^**adjusted OR = Odds ratio.

## Discussion

In this study, we sought to understand the basis for the long-term success of the MTB Uganda family in Kampala, Uganda. First, we examined the MTBC population structure and changes in prevalence of the different MTBC lineages circulating in Kampala during the last 18 years. Secondly, we investigated the risk factors associated with cavitary TB. Our data show that in peri-urban Kampala there are 3 dominant MTBC lineages causing TB, with L4-U being the most predominant and stable for the last 18 years, followed by the L4-NU and L3. Additionally, our multivariate results also showed that this long-term success of L4-U was not due to increased virulence when considering cavitary TB as a proxy, even though cavitary TB per se was independently associated with other known risk factors such as smoking, low socio-economic status and HIV-negative status [37-42]. We also note that, from out recruitment criteria only smear positive patients were enrolled as index probably this could have reduced our chances of finding any association of MTB lineage with cavitary TB. Nevertheless, among the contacts 80 % of the patients were smear negative and still this category of patients showed no association of MTB Uganda family with cavitation in a univariate analysis (data not shown).

### Prevalence of MTB lineages over time in peri-urban Kampala

Our data reiterate earlier findings that reported dominance of L4-U in Uganda [7,8,20]. In addition, the large sample size and longitudinal nature of our sample allowed us to investigate trends over time. We found that the dominance of L4-U over other Lineage 4 strains and Lineage 3 has been constant for at least the last 18 years, even with episodes of HIV and different interventions such as introduction of anti-retrovirus drugs and promotion of condom use. Taken together, these results are in agreement with the global phylogeography of MTBC, and show that the association between particular MTBC lineages and specific geographic settings is long-standing [2,3,6].

### Phenotypes of MTB lineages in peri-urban Kampala

There is increasing evidence from experiments in animal models and from clinical studies indicating that MTBC strains differ in their phenotypes [3,5,18]. For instance, the virulent H37Rv laboratory strain of MTBC induces less apoptosis in macrophages that the avirulent H37Ra strain [43]. Investigations in animal models have also indicated that virulence also varies between MTBC clinical strains [24,44-47]. Data from clinical cohorts have demonstrated that some strains are associated with fever [48], more severe disease [20], higher transmissibility [49], increased ability to progress from latency to active disease [50], extra pulmonary disease [51-53], and HIV co-infection [1,28]. Given that background, in this study, we tested the hypothesis that increased virulence could be responsible for the long-term success of L4-U in Kampala. We defined virulence as the ability of MTB lineage to cause cavitary TB, hence causing severe disease which is more transmissible [36,54]. Our data showed that all lineages identified in this study area had comparable odds of causing cavitary TB. This lack of association remained even after adjusting for socio-economic and patient clinical characteristics. However, this is contrary to an earlier smaller study that observed that L4-U was more virulent [20]. The fact that we identified other clinical risk factors previously shown to be associated with cavitary disease [37,55-61] serves as a “positive control” in our study, and supports our negative finding of the lack of association of MTBC lineage with cavitary disease.

### Association between MTBC variants and their human hosts

If the long-term dominance of L4-U is not due to increased virulence, what else could be at the basis of its success compared to other lineages in Kampala? MTBC is known to exhibit a strong phylogeographical population structure [1,28], which is also reflected at the “sub-lineage” level. For example, the co-called Cameroon family of MTBC is a sub-lineage of Lineage 4 and almost exclusively found in West-Africa [62]. Similarly, L4-U is mainly found in Uganda and neighboring countries, and rarely found elsewhere. Another important feature of the phylogeography of MTBC is that it remains stable even in large cosmopolitan settings such as San Francisco, London and Montreal, where at least some degree of intermingling between local and immigrant host and MTBC populations could be expected [1,6,26,63]. Moreover, several studies have shown that MTBC preferably transmits in sympatric host populations [1,28]. Based on these observations, it has been hypothesized that different MTBC lineages might have adapted to different human population, possibly as a consequence of the long co-evolutionary history of MTBC and its human host [1,2,5,6,10,28]. Our observation that L4-U has been dominating in Kampala for at least 2 decades is consistent with this hypothesis. However, much more work is needed to substantiate this notion, including studies that investigate the interaction between human and MTBC genetic diversity [27,64-67].

### Limitations of the study

First, this study defined disease outcome at presentation; this could have limited our ability to detect other parameters that contribute to disease outcome as the disease progresses. Second, our study was neither population-based nor completely random, possibly bringing about some selection bias. Nevertheless, the strength of our study is that it is based on patient information and laboratory data collected from two large, well-characterized and systematically followed up studies conducted over a long period of time.

## Conclusions

In summary, we observed 3 main MTBC lineages circulate in peri-urban Kampala, with the L4-U being predominant for the last 2 decades. We found that none of these MTBC lineages were associated with increased risk for cavitary TB.

## Abbreviations

MTBC: *Mycobacterium tuberculosis complex*; MTB: *Mycobacterium tuberculosis*; RD: Region of difference; OR: Odds ratio; aOR: Adjusted odds ratio; BCG: Bacillus Calmette-Guerin; JCRC: Joint Clinical Research Centre; NTRL: National Tuberculosis Reference Laboratory; TB: Tuberculosis; SNP: Single Nucleotide polymorphism; L4: *Mycobacterium tuberculosis* Lineage 4; L3: *Mycobacterium tuberculosis* Lineage 3; L4-U: *Mycobacterium tuberculosis* Uganda family; L4-NU: *Mycobacterium tuberculosis* lineage 4 other than Uganda family.

## Competing interest

The authors declare that they have no competing interest.

## Authors’ contributions

MLJ, SG and WHB conceived the idea; EW, MLJ, SG, WHB, KE. GK, SD designed and performed the experiments; EW, EM and SMD analyzed the data; EW, MLJ, SG, EM, SMD, GK, KE and WHB wrote the paper. All authors read and approved the final manuscript.

## Pre-publication history

The pre-publication history for this paper can be accessed here:

http://www.biomedcentral.com/1471-2334/13/484/prepub

## Supplementary Material

Additional file 1: Table S1 SNPs markers, primers and probes used in RT-PCR to genotype 1746 MTB isolates. Click here for file

Additional file 2: Figure S1 *M. tuberculosis* Uganda (L4-U) is the most prevalent MTBC lineage. Lineages of MTBC were identified based on the presence or absence of a defined SNP. To determine the presence or absence of each SNP, primers and hybridization probes were designed for use in a real-time PCR assay that distinguishes MTBC lineages based on differences in Tm (See Additional file 1: Table S1). Click here for file

Additional file 3: Figure S2 No trend from 1992–2009 for any MTBC lineage was observed. The MTBC lineage identification and proportions observed overtime is as described in legend of Additional file 2: Figure S1. The predicted frequencies of MTBC overtime were computed by Poisson logistic regression analysis. Click here for file
