# Supplementary material for: Long-term dominance of Mycobacterium tuberculosis Uganda family in peri-urban Kampala-Uganda is not associated with cavitary disease
Source: BMC Infect Dis. 2013 Oct 17;13:484. doi: 10.1186/1471-2334-13-484 (PMC3853102; doi:10.1186/1471-2334-13-484)
Supplement: Additional file 1: Table S1 — SNPs markers, primers and probes used in RT-PCR to genotype 1746 MTB isolates. [file 1471-2334-13-484-S1.doc]

**Tables**

**Supplementary Table 1: SNPs markers, primers and probes used in RT-PCR to genotype 1746 MTB isolates.**

| **SNP Name** | **Primer and probes** | **MTB lineage** | **Tm-1** | **Tm-2** | **Primer annealing temperature** |
| --- | --- | --- | --- | --- | --- |
| Rv2949c_0375s | Forward: 5-TTATAACAAGGTTGACGCAGACA-3 | (L4-U)-I | ٭58°C | 68°C | 53°C |
|  | Reverse: 5-CTCTCTTTCGGGAATTCTGATAC-3 |  |  |  |  |
|  | ACCTTCAAGGAAGAGGCTAAAGTCT-Fluo-3 |  |  |  |  |
|  | LC Red 640-GTCGGAGATTTGCCAGGTTGGCT-Phosphate |  |  |  |  |
| Rv0040c-0619n | Forward:5-ATT GCT CGA TGG CAG A-3 | (L4-U)-II | ٭62°C | 68°C | 57°C |
|  | Reverse: 5-AAA CCA GGT ACT TGT CGG-3 |  |  |  |  |
|  | LC Red 640-TGA TGA CGG AAA GCC GT**C** GAA A-Pho-3 |  |  |  |  |
|  | 5-GTT TTC GCG GTA GGT GCC CTC GAT G-Fluo-3 |  |  |  |  |
| Rv2962c-0711s | Forward: 5-GAA CGC CCT TTG CTC TTC-3 | L4-NU | ٭56°C | 64°C | 51°C |
|  | Reverse:5-CAA GGT ACT CGT GGT TGG-3 |  |  |  |  |
|  | LC Red 610- CCC GAG CTG ATG CCC ACC T-Pho-3 |  |  |  |  |
|  | 5-CAC ACC CTG TAT GC**C** GAC G-Fluo-3 |  |  |  |  |
| Rv0129c-0472n | Forward:5-CGA CTG GTA TCA GCC CTC-3 | L3 | ٭58°C | 68°C | 53° C |
|  | Reverse:5-GGA ACT GCT GCG GGT AGT A-3 |  |  |  |  |
|  | LC Red 610-GAC ACG **CCC** TTG TTG GCC-Pho-3 |  |  |  |  |
|  | 5-CGC CGC GTT GCC TGT CG –Fluo-3 |  |  |  |  |

Shaded and bold nucleotide denotes SNP position relative to H37Rv MTB genotype.

٭ Denotes MTB lineage specific melting temperature (Tm).

**Table 1: Distribution of patient variables across the 3 main MTB lineages in peri-ur**ban Kampala.

| **Variable** | **Category** | **Patients characteristics (n, %)** | **L4-U (n=788)** | **L4-NU (n=289)** | **L 3 (n=136)** | **P-value*** |
| --- | --- | --- | --- | --- | --- | --- |
| Age^1^ | > 30 years | 757 (69) | 503 (70) | 173 (66) | 81 (68) | 0.44 |
|  | ≤ 30 years | 340 (31) | 213 (30) | 89 (34) | 38 (32) |  |
| Sex^2^ | Female | 503 (46) | 325 (45) | 120 (46) | 58 (49) | 0.79 |
|  | Male | 594 (54) | 391 (55) | 142 (54) | 61 (51) |  |
| HIV status^3^ | Negative | 669 (65) | 450 (66) | 157 (62) | 62 (60) | 0.29 |
|  | positive | 367 (35) | 230 (34) | 95 (38) | 42 (40) |  |
| Cavity^4^ | No | 441 (47) | 296 (48) | 106 (48) | 39 (39) | 0.25 |
|  | Yes | 500 (53) | 324 (52) | 115 (52) | **61 (61)** |  |
| Ethnicity^5^ | Non-bantu | 64 (6) | 39 (6) | 14 (6) | 11 (9) | 0.27 |
|  | Bantu | 984 (94) | 641 (94) | 238 (94) | 105 (91) |  |
| Smoking^6^ status | Never smoked | 659 (62) | 443 (65) | 155 (60) | 61 (52) | **0.03** |
|  | Current or ever smoked | 401 (38) | 243 (35) | 102 (40) | 56 (48) |  |
| BMI ^7^ | Weight loss | 557 (49) | 374(50) | 121(44) | 62(48) | 0.19 |
|  | No loss | 588 (51) | 368(50) | 154(56) | 66(52) |  |
| Level of education ^8^ | Low | 377 (35) | 248 (36) | 93(36) | 36(31) | 0.52 |
|  | High | 688 (65) | 444(64) | 163(64) | 81(69) |  |
| Drinking alcohol ^9^ | Yes | 162 (21) | 106 (21) | 40(21) | 16(22) | 0.99 |
|  | No | 611 (79) | 402(79) | 151(79) | 58(78) |  |
| Income ^10^ | Low | 270 (46) | 162(44) | 69(46) | 39 (61) | **0.048** |
|  | High | 314 (54) | 203(56) | 82 (54) | 29 (39) |  |
| diabetic Patients ^11^ | Yes | 12 (1) | 8(2) | 3(2) | 1(2) | 0.97 |
|  | No | 922 (99) | 604(98) | 224(98) | 94(98) |  |
| Presence of BCG scar ^12^ | Yes | 756 (62) | 488 (62) | 183 (63) | 85 (63) | 0.92 |
|  | No | 456 (38) | 299 (38) | 106 (37) | 51 (38) |  |
| Night sweets^13^ | No | 533 (47) | 340(46) | 133(49) | 60 (47) | 0.69 |
|  | Yes | 608 (53) | 401 (54) | 139(51) | 68 (53) |  |
| TB in the past ^14^ | Yes | 17 (2) | 10 (2) | 3 (2) | 4 (4) | 0.18 |
|  | No | 953 (98) | 619 (98) | 239 (98) | 95 (96) |  |
| Hemoptysis^15^ | No | 173 (15) | 111(15) | 43(16) | 19 (15) | 0.95 |
|  | Yes | 972 (85) | 632(85) | 231(84) | 109 (85) |  |
| Swollen lymph nodes^16^ | No | 46 (6) | 26 (5) | 16 (8) | 4 (5) | 0.26 |
|  | Yes | 727 (94) | 482(95) | 175(91) | 70 (95) |  |
| Extent of lung involvement^17^ | Normal/mild | 733 (73) | 475 (72) | 180 (75) | 78 (76) | 0.58 |
|  | Advance/far advanced | 271 (27) | 185 (28) | 61 (25) | 25 (24) |  |
| Smear grade^18^ | ≤10 AFB/field | 227 (33) | 146 (33) | 65 (37) | 16 (24) | 0.1377 |
|  | >10 AFB/field | 454 (67) | 294 (67) | 109 (63) | 51 (76) |  |

*p-value obtained by chi-square statistic; 1 = 116 missed data for age, 2 = 116 missed data for sex, 3 = 177 missed data for HIV status, 4 = 272 missed data for cavity, 5 = 165 missed data for ethnicity, 6 = 153 missed data for smoking status, 7= 68 missed data for BMI, 8= 148 missed data for level of education, 9= 440 missed data for drinking alcohol, 10= 633 missed data for income, 11= 279 missed data for diabetes history, 12= 1 missed data for BCG status, 13 = 72 missed data for night sweating, 14= 243 missed data for history of TB, 15= 68 missed data for hemoptysis, 16 = 440 missed data for swollen glands, 17= 209 missed data for extent of lung involvement, 18=532 misses data of smear grade.

**Table.2:** A**ssociation between MTB lineages and patient characteristics with cavitary TB.**

| **Variable** | **Category** | **Odds Ratio©** | **95% CI** | **٭P-value** |
| --- | --- | --- | --- | --- |
| Lineage | Lineage L4-NU | 1 | - | - |
|  | Uganda( L4-U) | 0.90 | 0.69-1.18 | 0.45 |
|  | Lineage 3 | 1.43 | 0.94-2.19 | 0.10 |
| Age | > 30 years | 1 |  |  |
|  | ≤ 30 years | 1.13 | 0.85-1.49 | 0.40 |
| Sex | Female | 1 |  |  |
|  | Male | 1.24 | 0.96-1.60 | 0.10 |
| HIV status | Negative | 1 |  |  |
|  | Positive | 0.78 | 0.596-1.03 | 0.08 |
| Smoking status | Never smoked | 1 |  |  |
|  | Current or ever smoked | 4.78 | 3.41-6.69 | **<0.0001** |
| Income | High | 1 |  |  |
|  | Low | 2.13 | 1.52-2.97 | **<0.0001** |
| Hemoptysis | Yes | 1 |  |  |
|  | No | 2.10 | 1.46-3.03 | **<0.001** |

*p-value obtained by logistic regression analysis; **©** Unadjusted OR = Odds ratio

**Table 3. Multivariate analysis determining independent risk factors for development of cavitary TB.**

| **Variable** | **Category** | **Odds Ratio®** | **95% CI** | **P-value٭** |
| --- | --- | --- | --- | --- |
| Lineage | Lineage L4-NU | 1 | - | - |
|  | Uganda ( L4-U) | 1.08 | 0.78-1.51 | 0.6422 |
|  | Lineage 3 | 1.32 | 0.78-2.22 | 0.296 |
| Age | > 30 years | 1 | - | - |
|  | ≤ 30 years | 0.866 | 0.63-1.20 | 0.38 |
| HIV status | Negative | 1 | - | **-** |
|  | Positive | 0.62 | 0.45-0.84 | **0.0023** |
| Smoking status | Never smoked | 1 | - | - |
|  | Current or ever smoked | 4.76 | 3.33-6.84 | **<0.0001** |
| Income | High | 1 |  |  |
|  | Low | 2.10 | 1.47-3.01 | **<0.0001** |
| Hemoptysis | No | 1 | - | **-** |
|  | Yes | 1.64 | 1.10-2.42 | **0.014** |

*p-value obtained by logistic regression analysis; **®**adjusted OR = Odds ratio
