# Supplementary material for: Long-term dominance of Mycobacterium tuberculosis Uganda family in peri-urban Kampala-Uganda is not associated with cavitary disease
Source: BMC Infect Dis. 2013 Oct 17;13:484. doi: 10.1186/1471-2334-13-484 (PMC3853102; doi:10.1186/1471-2334-13-484)
Supplement: Additional file 2: Figure S1 — M. tuberculosis Uganda (L4-U) is the most prevalent MTBC lineage. Lineages of MTBC were identified based on the presence or absence of a defined SNP. To determine the presence or absence of each SNP, primers and hybridization probes were designed for use in a real-time PCR assay that distinguishes MTBC lineages based on differences in Tm (See Additional file 1: Table S1). [file 1471-2334-13-484-S2.doc]

**Supplementary Fig. 1**
