# Supplementary material for: Long-term dominance of Mycobacterium tuberculosis Uganda family in peri-urban Kampala-Uganda is not associated with cavitary disease
Source: BMC Infect Dis. 2013 Oct 17;13:484. doi: 10.1186/1471-2334-13-484 (PMC3853102; doi:10.1186/1471-2334-13-484)
Supplement: Additional file 3: Figure S2 — No trend from 1992–2009 for any MTBC lineage was observed. The MTBC lineage identification and proportions observed overtime is as described in legend of Additional file 2: Figure S1. The predicted frequencies of MTBC overtime were computed by Poisson logistic regression analysis. [file 1471-2334-13-484-S3.doc]

**Supplementary Fig 2**
